# Kinetics of Hydrogen Generation from Oxidation of Hydrogenated Silicon Nanocrystals in Aqueous Solutions

**DOI:** 10.3390/nano10071413

**Published:** 2020-07-20

**Authors:** Gauhar Mussabek, Sergei A. Alekseev, Anton I. Manilov, Sergii Tutashkonko, Tetyana Nychyporuk, Yerkin Shabdan, Gulshat Amirkhanova, Sergei V. Litvinenko, Valeriy A. Skryshevsky, Vladimir Lysenko

**Affiliations:** 1 Faculty of Physics and Technology, AI-Farabi Kazakh National University, 71, AI-Farabi Ave., Almaty 050040, Kazakhstan; nanotechkz2012@gmail.com; 2 Institute of Information and Computational Technologies, 125, Pushkin Str., Almaty 050000, Kazakhstan; gulshat.aa@gmail.com; 3 Chemistry Department, Taras Shevchenko National University of Kyiv, Volodymyrska Street, 64, 01601 Kyiv, Ukraine; alekseev@univ.kiev.ua; 4 Institute of High Technologies, Taras Shevchenko National University of Kyiv, Volodymyrska Street, 64, 01601 Kyiv, Ukraine; anmanilov@univ.kiev.ua (A.I.M.); litvin608pv@gmail.com (S.V.L.); skrysh@univ.kiev.ua (V.A.S.); 5 Nanotechnology Institute of Lyon (INL), UMR CNRS 5270, INSA de Lyon, University of Lyon, 69621 Villeurbanne, France; s.tutashkonko@gmail.com (S.T.); tetyana.nychyporuk@insa-lyon.fr (T.N.); 6 Light Matter Institute, UMR-5306, Claude Bernard University of Lyon, 2 rue Victor Grignard, 69622 Villeurbanne, France; vladimir.lysenko@univ-lyon1.fr

**Keywords:** hydrogen generation rate, porous silicon nanopowder, nanosilicon oxidation, engineering of silicon nanoparticles

## Abstract

Hydrogen generation rate is one of the most important parameters which must be considered for the development of engineering solutions in the field of hydrogen energy applications. In this paper, the kinetics of hydrogen generation from oxidation of hydrogenated porous silicon nanopowders in water are analyzed in detail. The splitting of the Si-H bonds of the nanopowders and water molecules during the oxidation reaction results in powerful hydrogen generation. The described technology is shown to be perfectly tunable and allows us to manage the kinetics by: (i) varying size distribution and porosity of silicon nanoparticles; (ii) chemical composition of oxidizing solutions; (iii) ambient temperature. In particular, hydrogen release below 0 °C is one of the significant advantages of such a technological way of performing hydrogen generation.

## 1. Introduction

Hydrogen and fuel cell technologies have strong potential to play a significant role in new energy systems [1]. In view of energy storage purpose, hydrogen can be easily converted into electricity and heat. The amount of energy produced during hydrogen combustion is higher in comparison with any other fuel of the same mass [2]. However, hydrogen cannot be considered as an alternative fuel but rather an energy carrier requesting the consumption of a primary energy for its production. Due to this fact, the number of power-to-gas pilot plants producing hydrogen from fluctuating renewable power sources increases all over the world [3]. Besides, for the energy vector for electricity, mobility and heat, hydrogen can be also used as a raw material for the chemical industry or for the synthesis of various hydrocarbon fuels. Ongoing research is underway to develop environmentally friendly and economical hydrogen production technologies that are essential for the hydrogen economy. It ensures the gradual transition from the actual energy economy to a cleaner and sustainable energy future [4].

Water is an important source of hydrogen within the concept of sustainable development. The splitting of water molecules into hydrogen and oxygen can be performed by means of “green” technologies. For example, water electrolysis constitutes a suitable pathway to hydrogen production [1,5]. There are different technologies for electrolysis, including alkaline, proton-exchange membrane and high-temperature solid oxide processes. They form a basis of the power-to-gas systems converting excess electricity into hydrogen [3,5]. Water electrolysis is convenient to be used in combination with photovoltaics and wind energy as well as in direct relation with the availability of electricity [6,7,8].

The splitting of water molecules can also be provided by heat obtained from a nuclear reactor or a concentrating solar system, such as a solar tower, solar trough, linear Fresnel system or dish system [1]. The direct decomposition of water driven by heat can be released using thermocatalytic processes [9]. The most promising and useful approach for large-scale hydrogen production is based on thermochemical cycles—redox, sulfur-iodine, copper-chlorine and others [10]. However, the thermal technologies require complicated industrial equipment and are not useful for small-scale and portable applications.

Photocatalytic water splitting under visible light irradiation allows for obtaining hydrogen from water with the use of sunlight in the presence of a suitable catalyst reducing the high activation energy of water decomposition [11,12]. Photocatalysts are typically made of metal oxides, metal sulfides, oxysulfides, oxynitrides and composites thereof. Titanium dioxide, cadmium sulfide and graphitic carbon nitride are the most studied [13,14,15]. Metal ion implantation and dye sensitization are very effective methods to extend the activating spectrum to the visible range [16]. In recent years, many nanoparticles are synthesized by clean technologies utilizing plants and microbes. Water splitting, based on these nanoparticles, provides a blueprint to sustain the clean energy demands [17]. However, the number of visible light-driven photocatalysts is still limited, and there is no photocatalyst which could satisfy all necessary requirements [1,11].

Water splitting can be also released in a biological way. The absorption of light by microalgae or cyanobacteria results in the generation of hydrogen, known as biophotolysis [18]. Additionally, hydrogen can be produced from CO and H_2_O, implementing the biological water gas shift reaction catalyzed by photosynthetic and fermentative bacteria [19]. A high yield of hydrogen and a high production rate can be reached using biomass or biomass-derived chemicals as raw materials. The conversion of biomass is achieved through biological or thermochemical routes, such as fermentation, reforming, gasification or pyrolysis [20,21].

An inexpensive and simple way of hydrogen production is also implemented by hydrolytic systems. In particular, the reaction of water with metals is characterized by a redox potential below the level corresponding to H^+^→H2 transformation [22]. During the chemical reaction, hydrogen is released, and a metal hydroxide or oxide is formed. Among various available reactions, aluminum-water reactions in either alkaline or neutral solutions are most commonly used [23,24,25]. However, the existence of a solid oxide on the surface of aluminum particles prevents air and moisture from accessing the underlying metal. The use of strong bases, the application of high temperature or the activation of the aluminum metal [25,26,27] resolve this problem.

Metal hydrides are also efficient materials for the chemical decomposition of water. Indeed, in this case, not only the hydrogen liberated by oxidation of the corresponding metal is released, but also the hydrogen bonded in the hydride molecules. In particular, borohydrides are very promising materials, due to their very high theoretical capacities of hydrogen content. For example, sodium borohydride (NaBH4), lithium borohydride (LiBH_4_) and the ammonia–borane complex (NH_3_BH_3_) were widely investigated [1,22,28,29,30].

Recent reports devoted to the hydrolysis of magnesium composites (MgH_2_, Mg-oxide, Mg_2_Si, Mg-graphite, NdNiMg_15_) [21,22,23,24,25,26,27,28,29,30,31,32,33,34,35] and sodium metal [36] have demonstrated considerable hydrogen generation capabilities. However, the successful utilization of these materials in hydrogen-based energy systems is still quite expensive.

Silicon has also been investigated for hydrogen generation purposes [37]. Indeed, hydrogen can be produced, for example, from the reaction between ferrosilicon, sodium hydroxide and water. Further investigations showed the high potential and economic efficiency of the silicon-based hydrogen fuel [38,39]. In strong contrast to oil and molecular hydrogen, the transport and storage of silicon are free from potential hazards and require a simple infrastructure similar to that requested for coal. Whereas the latter material is converted to carbon dioxide, silicon involved in hydrogen production is transformed in sand. Among a number of the known metalloids, silicon shows the best efficiency for the chemical splitting of water [40,41]. Moreover, photoelectrochemical applications of crystalline silicon have been investigated for solar-induced splitting of water [42,43].

Silicon nanoparticles can be used to generate hydrogen much more rapidly than bulk silicon because of their high specific surface area. Chemical or anodic etching [44,45,46], the beads milling method [47,48,49], or laser pyrolysis [50] can produce the nano-powders used for hydrogen generation from oxidation reactions of Si in water. Since the reaction rate strongly depends on pH, the addition of alkalis or ammonia is often used in order to increase the rate of hydrogen production.

The fabrication of silicon nanostructures by electrochemical etching has a significant advantage over the other approaches cited above. Indeed, it results in the formation of nano-porous morphologies covered with an abundant number of silane groups (SiH_X_) [51,52,53]. The maximum specific surface of nanoporous silicon can reach the value of 800 m^2^/g, while the content of hydrogen chemically bound on the surface can be as high as 60 mmol of atomic H per gram of porous Si, corresponding to the H/Si ratio ~1.8 or to the 6% mass of H [44]. The presence of this surface hydrogen can increase the output volume of H_2_, which releases in the reaction of porous silicon nanoparticles with water by 1.5 times [54]. General hydrogen output and release rate strongly depend on the porosity and sizes of the particles. Moreover, sufficient reaction rates are achieved even at room temperature, without additional heating or mixing [55,56]. A working prototype of cartridge generating hydrogen from PSi nanopowder was designed and coupled with a portable fuel cell [57].

Silicon nanostructures have also been considered as good candidates for photoelectrochemical and photocatalytic water splitting to produce hydrogen. Photo-electrodes based on arrays of silicon nanowires coupled with different catalysts [58,59,60], as well as porous silicon structures [61,62,63], were used for the generation of hydrogen. The surface modification of silicon nanostructures plays a considerable role in designing materials for solar-driven catalysis. The applications of Si nanostructures can be moved from photoelectrical to photochemical conversion by taking catalytic sites into account [58]. However, the chemical reactivity of silicon surface with water complicates the implementation of the photocatalytic reaction. This is especially evident for the porous nanostructures, where a higher oxidation degree of nanosilicon can result in the blocking of the nanopores with silicon oxide [64].

## 2. Hydrogen Generation from Oxidation of Porous Silicon Nanopowders in Water

Research work described in the current paper is devoted to the investigation of influence of various physico-chemical factors on the kinetics of hydrogen generation resulting from chemical reactions of hydrogenated porous silicon (PSi) nanopowders with water. In particular, the impact of ambient temperature, chemical nature and the concentration of used alkalis, grinding degree, and the porosity of PSi nanopowders are examined. In general, this reaction occurs as follows:

(1) $$SiH_{X}+3H_{2}O\to SiO_{2}•H_{2}O+\left(2+\frac{X}{2}\right)H_{2}↑+Q$$

where *Q* is equal to 361 kJ/M and to 634 kJ/M for *x* = 0 and *x* = 2, respectively.

**Figure 1:**
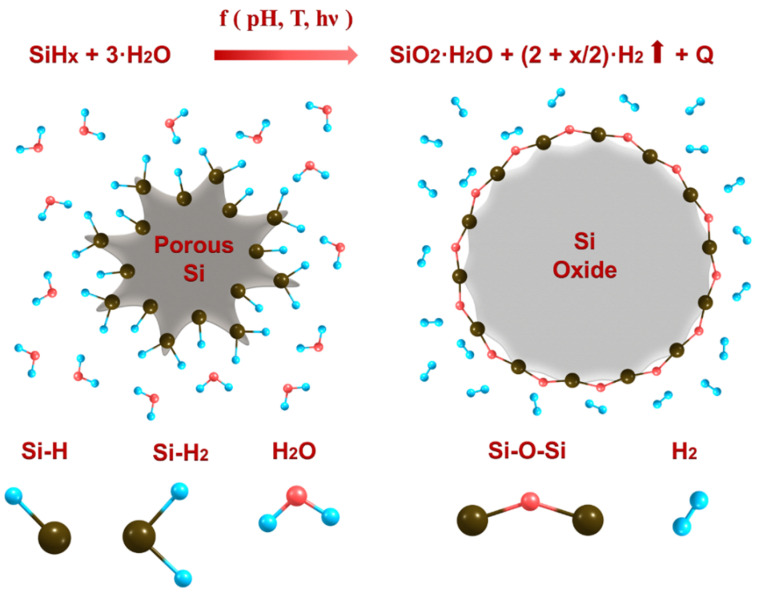
Transformation of partially hydrogenated PSi nanoparticles in silicon oxide in water solution.

Reaction (1) is schematically illustrated in Figure 1.

As one can see, partially hydrogenated PSi nanoparticles are almost completely transformed in silicon oxide nanoparticles, as shown in Figure S1 in the Supplementary Materials. This transformation is modulated by the pH and temperature of the surrounding aqueous solution, as well as by the intensity of external light results in the abundant production of molecular hydrogen [44,45].

**Figure 2:**
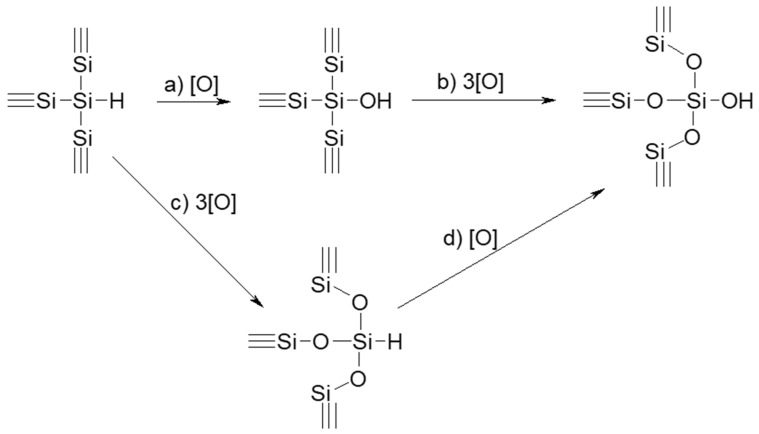
Schematic representation of gradual transformation of initially hydrogenated PSi nanopowder in silicon oxide during its oxidation in water.

Si-H bonds covering specific surface of PSi nanoparticles are known to be the most chemically active ones. These bonds can react, for example, with the bases, as shown by reaction (a) in Figure 2, substituting surface hydrogen by –OH groups. Si–Si bonds located on the PSi surface can also react with oxidizers, according to the reactions (b) and (c) in Figure 2, leading to the formation of O_3_Si–H and Si–OH surface fragments. As a result, a silicon oxide film starts to appear on the PSi surface. The O_3_Si–H fragments are rather passive and stable. Nevertheless, they can react with strong bases, as shown by reaction (d) in Figure 2 [44,54].

Reactivity of the chemical bonds of PSi surface with water and other oxidants increases in the following order: O_3_Si–H < Si_3_Si–H < Si–Si. In the other words, the oxidation of the Si surface proceeds more preferably via (c)–(d), than via (a)–(b), as shown in Figure 2 [45,65,66]. When a sufficiently thick layer of hydrated silicon oxide is formed on the PSi surface, it efficiently preserves the inner parts of Si NPs from further oxidation, and the reaction stops [40]. The presence of bases in aqueous solution results in the significant acceleration of the reaction between Si and water due to an increase in the primary reaction rate of the surface bonds with water as well as due to the partial dissolution of the surface oxide layer and penetration of water molecules and OH^–^ ions to the inner parts of the PSi nanoparticles.

## 3. Materials and Methods

### 3.1. Formation of PSi Nanopowders

The PSi nanopowders used in this study were obtained by mechanical grinding of porous silicon (PSi) layers. The initial PSi layers were fabricated according to a standard procedure based on electrochemical etching [67] of monocrystalline (100)-oriented boron-doped Si wafers (1–10 Ω cm and 0.01 Ω cm for nano- and meso-PSi structures, respectively) at current densities of 2–340 mA/cm^2^. The etching solutions of 9:1, 3:1 and 1:1 mixtures (by volume) of concentrated aqueous hydrofluoric acid (48%) and ethanol were used for nano-PSi formation and an HF/ethanol solution of 1:1 volume mixture for meso-PSi formation. The experimental conditions of the electrochemical etching mentioned above allowed for the formation of nano-PSi and meso-PSi nanopowders with various porosities, as shown in see Figures S2–S4 in Supplementary Materials, and sizes of nanoparticles constituting the porous powders, as shown in see Figure S5 in Supplementary Materials.

### 3.2. Structural Characterization of PSi Nanopowders

Structural properties and morphologies of the PSi nanopowders were investigated by means of atomic force microscopy (AFM), scanning electron microscopy (SEM) and transmission electron microscopy (TEM). Atomic force microscopy (AFM) characterization was performed on the Digital Instruments 3100 instrument using ultra-sharp silicon cantilevers (Nanosensors^TM^ SSS-NCH, Switzerland) with a typical curvature radius of 10 nm and nominal spring constant of 42 N/m. AFM images were acquired in a tapping mode at room temperature under ambient conditions for the particles deposited onto an atomically flat surface of electronic grade silicon wafers. Scanning electron microscope (SEM) images were taken using the ultra-high resolution Tescan MIRA 3 scanning electron microscope.

### 3.3. Temperature-Programmed Desorption (TPD)

The TPD studies were performed in a quartz reactor, in the range of 40–1000 °C by means of a Thermoquest TPD/R/O 1100 analyzer. A quadruple mass analyzer (Quadstar 422, Baltzer, Germany) coupled to the TPD apparatus allows for the identification of desorbed species. To obtain quantitative measurements on the molecular hydrogen desorbed from the sample surface, calibration was performed by injecting known amounts of hydrogen into the argon flow and evaluating the area of the detected TPD signal. Figure S6 in the Supplementary Materials shows typical TPD signals obtained on nano- and meso-PSi powders. Comparison between the TPD curves and mass spectrum of the desorbed species indicates that the desorbed phase corresponds perfectly to H_2_. These results prove the presence of hydrogen in the initial as-prepared PSi nanopowders before their reaction with oxidizing aqueous solutions. The integration of the shown TPD signals allows us to estimate hydrogen quantity in the PSi nanopowders. In particular, nano-PSi powder can contain hydrogen with quantities more than one order of magnitude compared to the meso-PSi samples. It can be explained by the much more important specific surface area of the nano-PSi powder, which can exceed 1000 m^2^ g^−1^ [67].

### 3.4. Measurement of Hydrogen Generation Kinetics

All of the experiments, aimed at the recording of hydrogen generation kinetics during the reaction (1), were carried out on a working system, shown in Figure S7 in Supplementary Materials. A Plexiglas container was used as the reaction chamber. Samples of the PSi nanopowders were put on a rotating sample holder fixed in the upper part of the Plexiglas container. The weight of the used PSi samples varied from 50 to 100 mg. The volume of the oxidizing solutions was 20 mL. The PSi nanopowders were immersed into the oxidizing solution by rotation of the sample holder. Hydrogen generation started immediately after the beginning of reaction (1). Generated hydrogen was accumulated in the upper part of a graduated glass tube allowing for the measurement of the hydrogen volume. Systematic errors, caused by excess pressure of the superseded water, evaporation of the solution, and solubility of hydrogen in water, are estimated to be inessential (<10%) and were not taken into account. The experiments were carried out at low ambient temperature (−40 °C) and at room temperature (+23 °C) for 160 h and 3 h, respectively.

## 4. Results and Discussion

**Figure 3:**
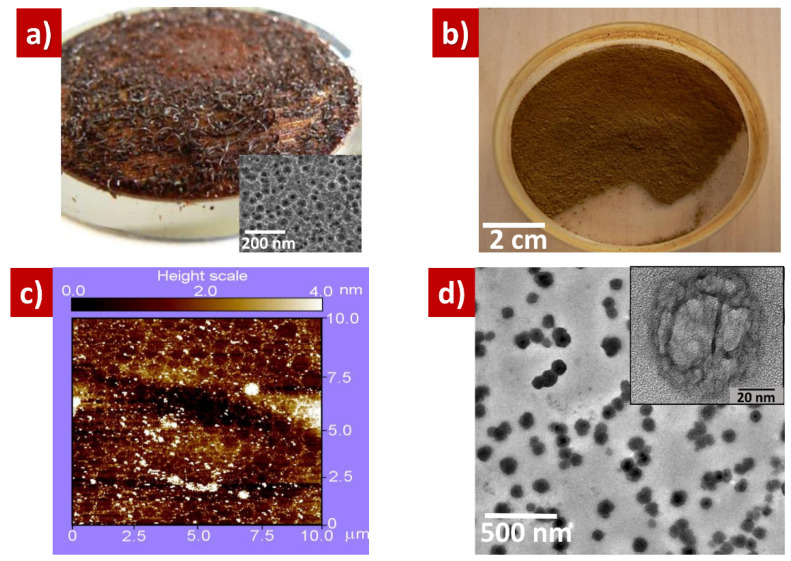
(**a**) As-prepared hydrogenated highly porous silicon film, inset: SEM top view image of the nanoporous Si film; (**b**) PSi nanopowder obtained by mechanical grinding of the PSi layer; (**c**) AFM image of the PSi nanoparticles constituting the PSi nanopowder; (**d**) TEM images of the PSi nanoparticles, inset: single PSi nanoparticle.

Typical view of an as-prepared hydrogenated highly porous cracked silicon film and its SEM top image (in inset) are shown in Figure 3a. The porous film is easily auto-destroyed during its drying in ambient air after formation by electrochemical etching. To obtain porous Si (PSi) nanopowders, the porous films were additionally mechanically grinded, as shown in Figure 3b. The AFM picture shown in Figure 3c confirms the nano-scale size range of the nanoparticles (white spots and points), constituting the PSi nanopowder. The TEM image of the porous nanoparticles, presented in Figure 3d, allows us to estimate their sizes (50–60 nm) as well as clearly reveals their porous morphology (see inset illustrating a single PSi nanoparticle). Porosity values of the PSi nanoparticles used in our experiments were in the range of 30–95%. Their pore sizes varied from several up to 20 nm and specific surface area was between 100–800 m^2^ g^−1^ [68].

**Figure 4:**
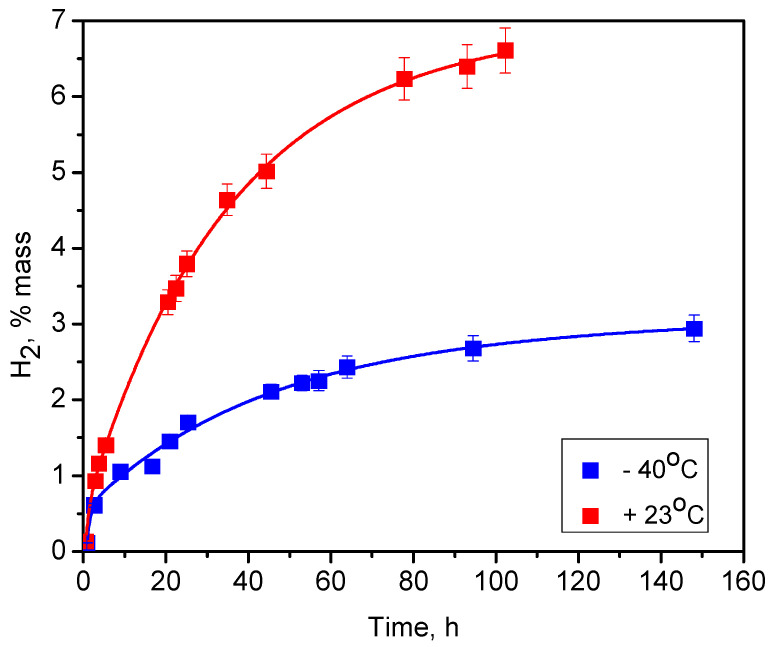
Kinetics of hydrogen generation due to oxidation reaction of meso-PSi nanopowders in water:ethanol solutions at different temperatures. Discrete points correspond to experimentally measured values, while solid lines are the fitting functions given in Supplementary Materials. The values on y-axis correspond to the percentage mass of H_2_ related to the mass of the reacted PSi nanopowder and water, which is necessary for the reaction.

Figure 4 shows the kinetics of hydrogen generation due to the oxidation of meso-porous silicon powders in water:ethanol = 3:2 solutions at two ambient temperatures: +23 °C and −40 °C. Ethanol was added to avoid the crystallization of water at negative temperatures as well as for the wetting of the PSi nanoparticles surface, because fresh as-prepared PSi nanopowder is hydrophobic. In both cases, the overall reaction time exceeded 100 h. It is worth remarking that hydrogen generation occurs even at relatively low temperatures (<0 °C) and this fact has a huge practical importance if one uses this reaction for hydrogen generation at negative temperatures. Since the chemical oxidation of silicon in aqueous solutions is thermally activated, a significant increase in H_2_ generation rate (factor of three and final global quantity (more than 2-times) of the generated hydrogen can be observed at the higher temperature.

The observed difference between the presented curves describing the behavior of the hydrogen generation kinetics at −40 °C and +23 °C can be completely explained by taking into account the temperature-dependent diffusion coefficient of the oxidizing water molecules through the silicon oxide progressively formed at the nanoparticle surface. Indeed, at the lowest temperature (−40 °C), a relatively low value of the diffusion coefficient limits the oxidation reaction rate and, consequently, hydrogen generation kinetics. In addition, the water molecules cannot wade through the formed oxide sheet of a certain thickness and it leads only to a partial oxidation of the silicon nanoparticles and limits the overall quantity of the generated hydrogen at a level of 2.8% mass. The water diffusion coefficient increases at the higher temperature (+23 °C) and it automatically leads to the enhanced hydrogen generation kinetics, as well as to the much higher quantity of hydrogen (6.7% mass) generated at the end of the reaction between the silicon nanopowder and water/ethanol mixture.

**Figure 5:**
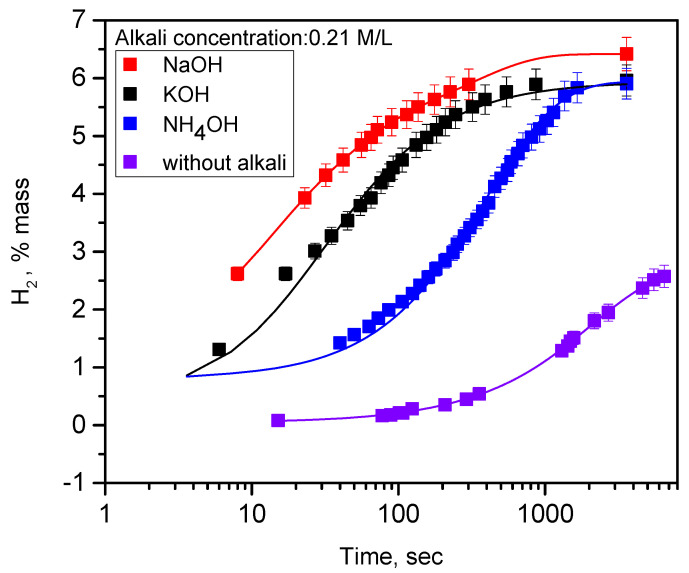
Kinetics of hydrogen generation due to oxidation reaction of meso-PSi nanopowders in water:ethanol:alkali solutions at room temperatures. Concentration of the used alkalis was 0.21 M/L. Discrete points correspond to experimentally measured values, while solid lines are the fitting functions given in Supplementary Materials.

Since alkali is known to accelerate the dissolution of silicon, hydrogen generation resulting from the oxidation of meso-PSi powders was studied in water:ethanol solutions containing alkali. Figure 5 allows us to compare the hydrogen generation kinetics, depending on chemical nature of different alkalis at same concentration of 0.21 M/L. As one can see, the presence of any type of alkali accelerates significantly (by several orders of magnitude) the hydrogen generation rate. Among the used alkalis, the relatively weak NH_3_ base demonstrates the slowest H_2_ generation. The higher H_2_ generation rate for NaOH in comparison with KOH is probably due to the better permeability of the surface oxide layer to smaller Na^+^ ions than for larger K^+^ ions and, as a result, the better access of active OH– ions to the SiO_2_/Si interface. At the same time, the final quantity (6% mass) of the generated hydrogen is similar for all the used oxidizing solutions and it is very close to theoretical value (6.25%). White color and the absence of visible photoluminescence of all the products formed in the reaction of PSi nanopowders with aqueous alkaline solutions at room temperature, confirm the total oxidation of the Si phase. Indeed, it can be easily understood, considering that hydrogen generation stops when silicon nanoparticles are completely oxidized.

**Figure 6:**
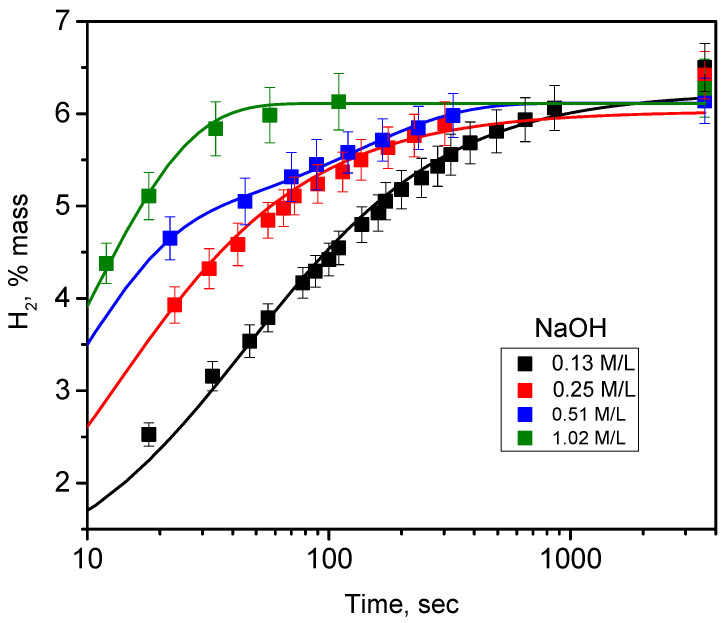
Kinetics of hydrogen generation due to oxidation reaction of meso-PSi nanopowders in water:ethanol:NaOH solutions at different concentrations of the alkali. Discrete points correspond to experimentally measured values, while solid lines are the fitting functions given in Supplementary Materials.

Since NaOH was found to be the most efficient alkali accelerating hydrogen generation from the oxidation of PSi nanopowders in aqueous solution, it was used for further additional studies. In particular, the impact of NaOH concentration on hydrogen generation kinetics was studied and is shown in Figure 6. As one can see, the higher the alkali concentration is, the higher the hydrogen generation rate is, as shown in Figure S8 in Supplementary Materials. Once again, the overall hydrogen generation quantity is independent on the alkali concentration. Finally, as shown in Figure S8 in Supplementary Materials, the same kinetics of hydrogen generation can be achieved at smaller concentrations of NaOH compared to KOH.

**Figure 7:**
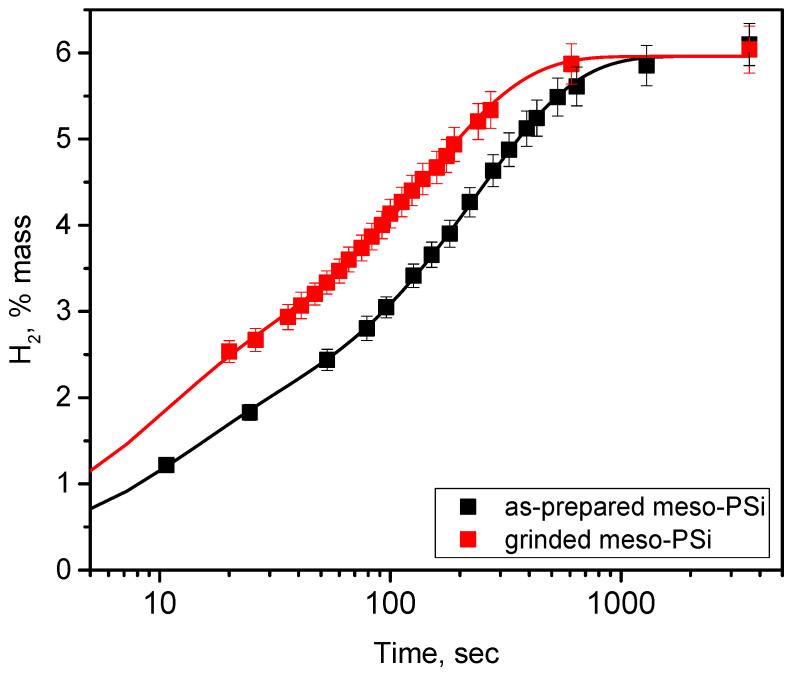
Kinetics of hydrogen generation depending on grinding degree of meso-PSi nanopowders. The oxidation reaction takes place in solutions of water:ethanol = 3:2 containing NH_4_OH in a concentration of 0.77 M/L. Discrete points correspond to experimentally measured values, while solid lines are the fitting functions given in Supplementary Materials.

Kinetics of hydrogen generation depending on the grinding degree of meso-PSi nanopowder are shown in Figure 7. Two different nanopowder morphologies—as prepared meso-PSi and grinded meso-PSi—shown in Figure 3a,b, respectively, were used. It can be seen that oxidation of the grinded meso-PSi accompanied by formation of hydrogen goes two-times faster in comparison with the as-prepared meso-PSi (analytical expressions of fitting functions can be found in Supplementary Materials. Such behavior of the hydrogen generation kinetics can be explained by much smaller sizes of nanoparticles (50–200 nm) of the grinded meso-PSi compared to the as-prepared powder constituted by porous particles from 100 μm–1 mm range.

**Figure 8:**
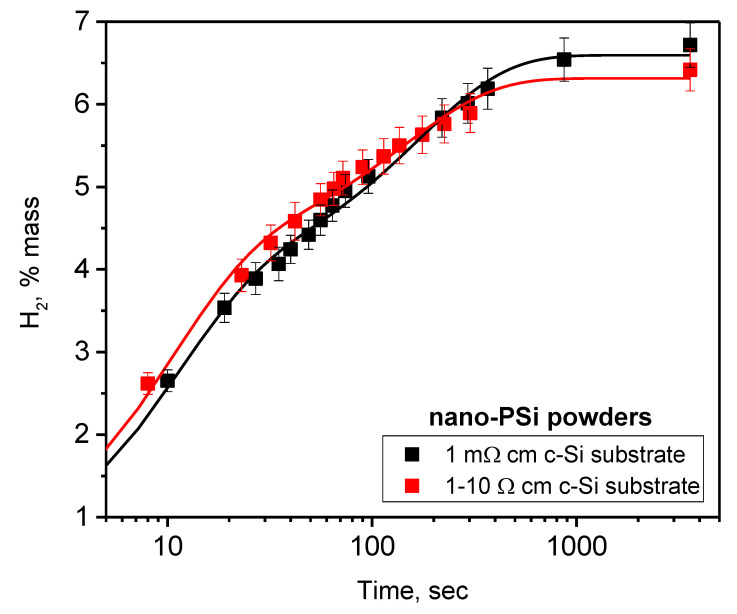
Kinetics of hydrogen generation depending on resistivity of silicon substrates used for fabrication of nano-PSi powder. The oxidation reaction occurs in solutions of water:ethanol = 3:2 containing NaOH in concentration of 0.21 M/L. Discrete points correspond to experimentally measured values, while solid lines are the fitting functions given in Supplementary Materials.

Hydrogen generation kinetics characterizing chemical reaction between aqueous alkaline solution and grinded nano-PSi powders obtained from c-Si substrates with low (ρ = 1–10 Ω·cm) and high (ρ = 1 Ω·cm) resistivities are compared in Figure 8. As one can see, the resistivity of the initial silicon substrate does not affect the kinetics of hydrogen release. Thus, only the nanoscale morphology of the nano-PSi powder (similar for the both resistivity levels), shown in Figures S2 and S4 in Supplementary Materials, determines the hydrogen generation kinetics.

**Figure 9:**
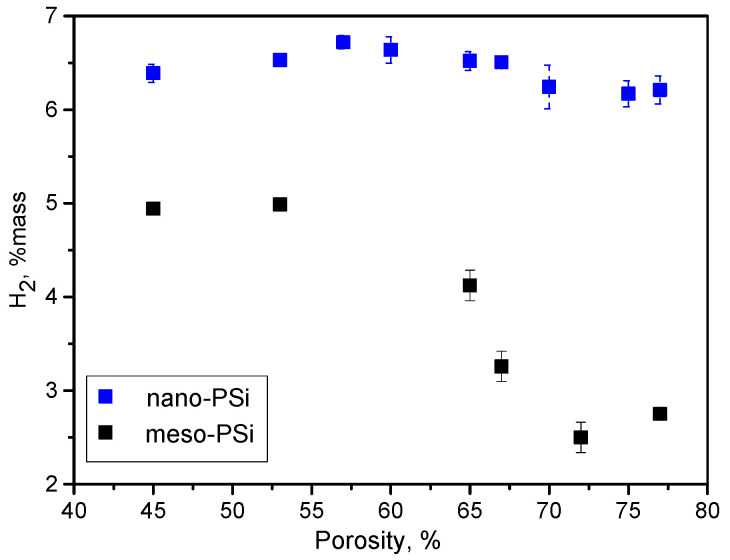
Porosity dependent hydrogen generation amount due to oxidation reaction of meso- and nano-PSi powders in H_2_O:Ethanol:NaOH solutions at room temperature. Alkali concentration was 0.5 M/L.

Figure 9 shows dependence of the global amount of generated hydrogen on porosity of the used meso-PSi and nano-PSi powders. In the case of nano-PSi powder, the quantity of the generated hydrogen is porosity-independent, while a significant decrease in hydrogen quantity was observed for higher porosity values of meso-PSi powder. This difference can be explained by smaller nanoparticle size and larger pores for nano-PSi in comparison to meso-PSi, as shown in Figure S5 in Supplementary Materials. Indeed, the hydrogen generation efficiency will be enhanced if the oxidizing solution easily in penetrates nanopores and completely oxidizes the smaller nanoparticles. In addition, the general behavior of the dependence of hydrogen amount on porosity for both the morphologies correlate perfectly with evolution of nanocrystals of the corresponding powders, as shown in Figure S5 in Supplementary Materials.

## 5. Conclusions

Hydrogen generation rate is one of the most important parameters which must be considered for the design of engineering solutions for hydrogen energy applications. Indeed, sometimes a slow and long-term production of hydrogen is requested, while for other kinds of applications the instantaneous generation of a very large amount of hydrogen must be provided. For the given technological approach, various parameters should allow for the control of the hydrogen generation rates in as large a range as possible. In our paper, precise experimental conditions, allowing the tuning of the hydrogen generation process based on the oxidation reaction of porous silicon nanoparticles in aqueous solutions, are reported. The hydrogen generation rate is dependent on chemical composition and the concentration of alkali added in the oxidizing solutions. In particular, the higher the alkali concentration is, the higher the hydrogen generation rate is, while the global amount of the released hydrogen remains constant. The size distribution of the porous silicon nanopowder also affects the generation rate values of produced hydrogen. For example, the smaller the nanoparticle sizes are, the more intense the oxidation reaction and, consequently, the higher the hydrogen generation rates are. Finally, hydrogen release below 0 °C is one of the significant advantages of such a technological way of hydrogen generation in comparison with numerous other developed technologies reported earlier. The reported experimental results confirm a huge technological potential of the hydrogen generation based on the splitting of Si–H bonds of porous silicon nanopowders and water molecules during the oxidation reaction.

## Supplementary Materials

The following are available online at https://www.mdpi.com/2079-4991/10/7/1413/s1, Figure S1: Photos of initial partially hydrogenated PSi nanopowder (before its reaction with water) and silicon oxide nanopowder obtained after hydrogen generation from oxidation of the PSi nanopowder in aqueous solutions, Figure S2: Nano-PSi: porosity as a function of the current density for three different HF concentrations (18% (HF:Ethanol = 1:1.66), 24% (1:1), 36% (3:1)), thickness of the porous layer is 7 μm, initial substrate was p-type Si (ρ~1–10 Ω·cm), Figure S3: Meso-PSi: porosity as a function of the current density for three different HF concentrations (18% (HF:Ethanol = 1:1.66), 24% (1:1), 36% (3:1)), thickness of the porous layer is 12 μm, initial substrate was p+- type Si (ρ~10-50 Ω·cm), Figure S4: Nano-PSi: porosity as a function of the current density for three different HF concentrations (18% (HF:Ethanol = 1:1.66), 24% (1:1), 36% (3:1)), thickness of the porous layer is 12 μm, initial substrate was p++- type Si (ρ~1–3 mΩ·cm), Figure S5: Dimentions of silicon nanocrystalls as function of porosity for the meso- and nano-PSi powders, Figure S6: Effusion curves for H2 desorption from the nano- and meso-PSi powders, Figure S7: Photo of the working system used for measurements of hydrogen generation kinetics, Figure S8: Hydrogen generation rates deduced from experimental the curves shown in Figure 5 and Figure 6, Figure S9: Hydrogen amount in terms of %mass (%m) versus the ratio between mass of the oxidizing solution (msol) and mass of meso-PSi nanopowder (mPS). Table S1: Comparison of the hydrogen generation methods, Table S2: Data summarizing the chemical reactions used to produce H2.
