# Supplementary material for: Kinetics of Hydrogen Generation from Oxidation of Hydrogenated Silicon Nanocrystals in Aqueous Solutions
Source: Nanomaterials (Basel). 2020 Jul 20;10(7):1413. doi: 10.3390/nano10071413 (PMC7408030; doi:10.3390/nano10071413)
Supplement: Supplementary file 1 [file nanomaterials-10-01413-s001.pdf]

Supplementary Materials

Kinetics of hydrogen generation from oxidation of hydrogenated silicon nanocrystals in aqueous solutions

Gauhar Mussabek, Sergei A. Alekseev, S. Anton I. Manilov, Sergii Tutashkonko, Tetyana Nychyporuk, Yerkin Shabdan, Gulshat Amirkhanova, Sergei V. Litvinenko, Valeriy A. Skryshevsky and Vladimir Lysenko

Table S1. Comparison of the hydrogen generation methods.

№	**Hydrogen generation method**	**Advantages**	**Drawbacks**
1	Electrolysis of water	The technology is convenient to be used in combination with photovoltaics and wind energy.<br>There are commercial units available for high power and portable applications.	Primary sources of electricity are needed.<br>Operating costs are especially much higher than for the thermochemical routes.
2	Thermocatalytic water splitting and thermochemical cycles	The methods have potential to be performed with solar heat and geothermal resources.<br>The commercial plants for mass production are expected.	Complicated industrial equipment is required that is not useful for small-scale and portable applications.<br>The feasibility of the cycles is typically linked to nuclear power.
3	Photocatalytic water splitting	Direct conversion of solar energy to hydrogen production is released.<br>The light-induced tunable technology allows on-demand generation.	A stable photocatalyst absorbing a wide range of solar radiation is needed.<br>The quantum efficiency and the rate of hydrogen production have low values.
4	Biological water splitting and biomass-derived hydrogen	Renewable raw materials are used.<br>Bio-hydrogen can be produced at a high yield or a high production rate.<br>Municipal wastes, crops wastes and industrial wastes can be used as alternative feedstocks	Life cycle assessment for the biological technologies is still a hard task.<br>An additional purification of the produced gas is required.<br>Pretreatment of biomass is needed.
5	Hydrolytic reactions of metals with water	The technology is simple and relatively flexible.<br>The raw materials are relatively cheap.	Strong bases and high temperatures are needed.<br>The reaction is characterized by long inductive times blocking hydrolysis initiation.<br>The additional pretreatment is often required to activate the material.

6	Hydrolysis of metal hydrides	The reaction provides high rates of hydrogen generation.<br>The process is characterized by a high hydrogen mass% value.	Hydrides are expensive, so they are more attractive for reversible hydrogen storage than for hydrogen production.<br>The chemical reaction of metal hydrides with water is often accompanied by toxic products.
7	Hydrolysis of silicon nanomaterials	The material is biocompatible.<br>The technology is cheap and flexible.<br>The production of hydrogen is possible at room temperature and normal conditions.	Silicon raw material should be transformed into nanopowder by the additional process.<br>Bases are needed for acceleration of the hydrogen generation.

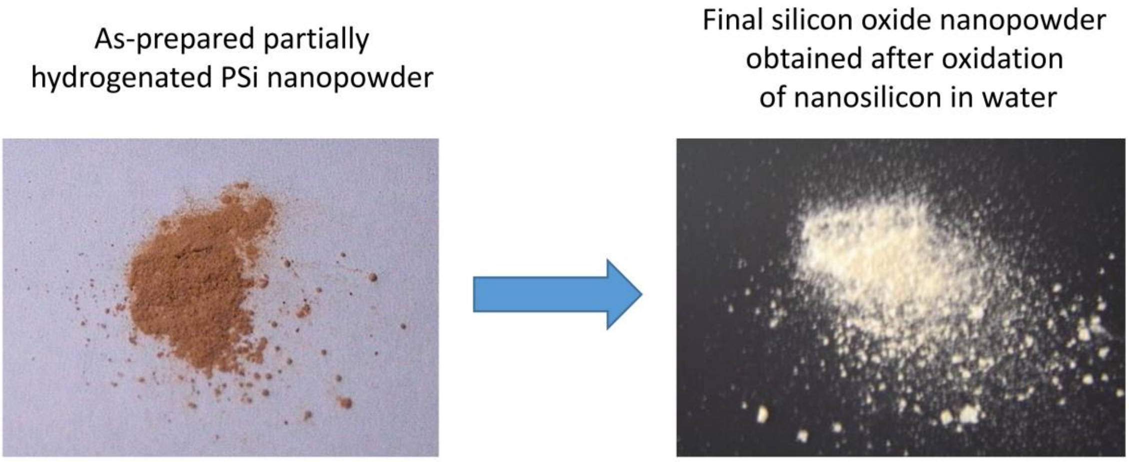

Figure S1. Photos of initial partially hydrogenated PSi nanopowder (before its reaction with water) and silicon oxide nanopowder obtained after hydrogen generation from oxidation of the PSi nanopowder in aqueous solutions

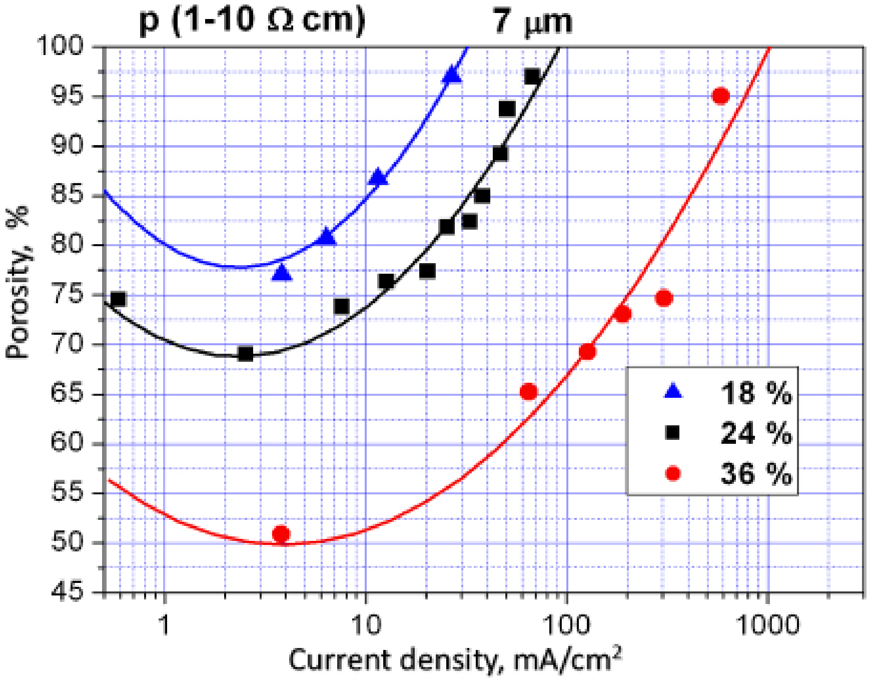

Figure S2. **Nano-PSi :** porosity as a function of the current density for three different HF concentrations (18% (HF: Ethanol = 1: 1.66), 24% (1: 1), 36% (3: 1)), thickness of the porous layer is 7 μm, initial substrate was p-type Si (ρ~1-10 Ω.cm).

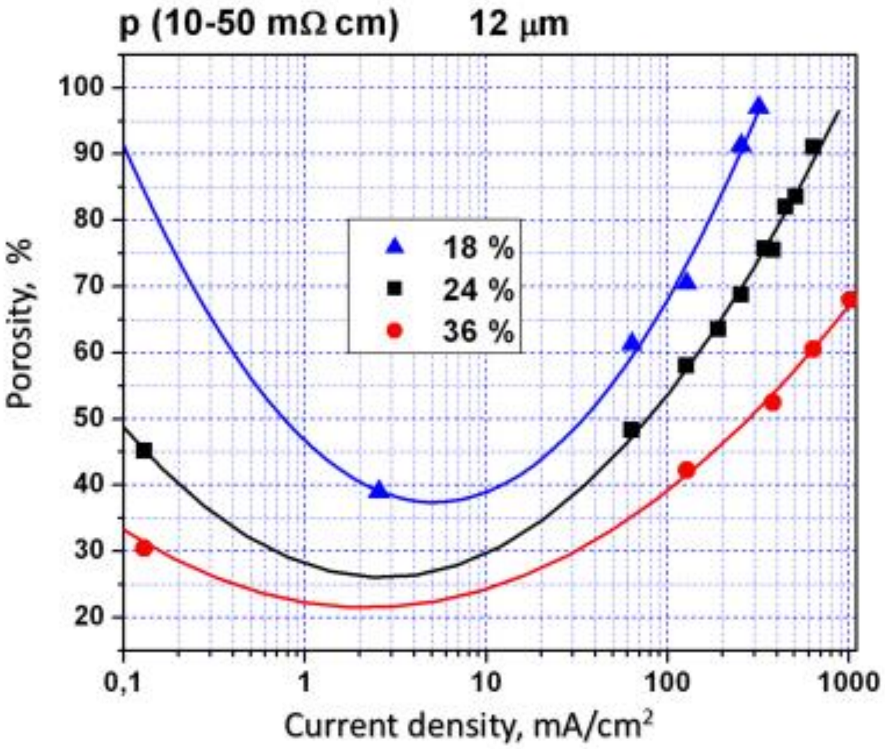

Figure S3. **Meso-PSi :** porosity as a function of the current density for three different HF concentrations (18% (HF: Ethanol = 1: 1.66), 24% (1: 1), 36% (3: 1)), thickness of the porous layer is 12 μm, initial substrate was p^+- type Si (ρ~10-50 Ω.cm).

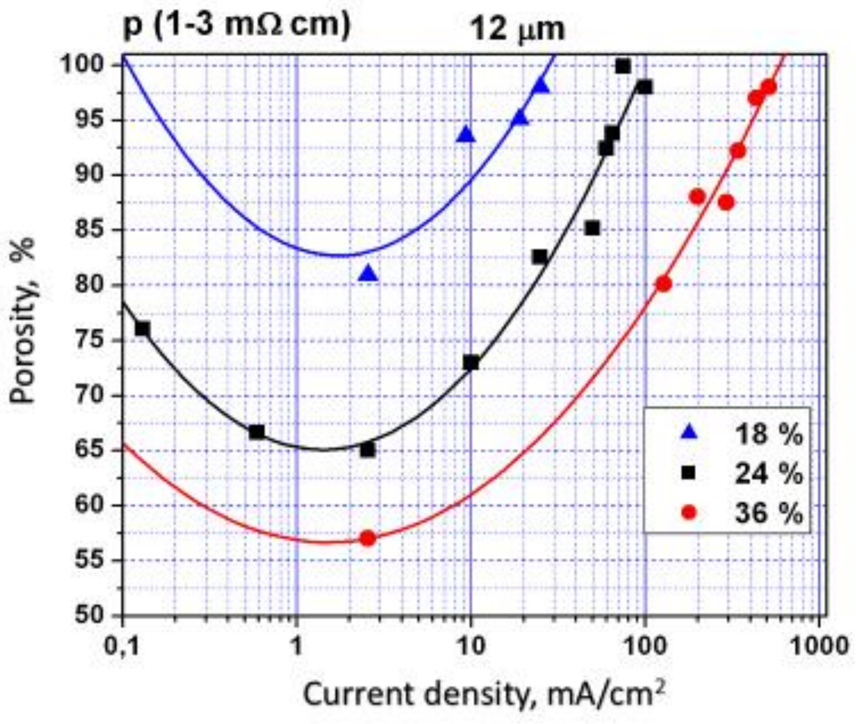

Figure S4. **Nano-PSi :** porosity as a function of the current density for three different HF concentrations (18% (HF: Ethanol = 1: 1.66), 24% (1: 1), 36% (3: 1)), thickness of the porous layer is 12 μm, initial substrate was p^{++}- type Si (ρ~1-3 mΩ.cm).

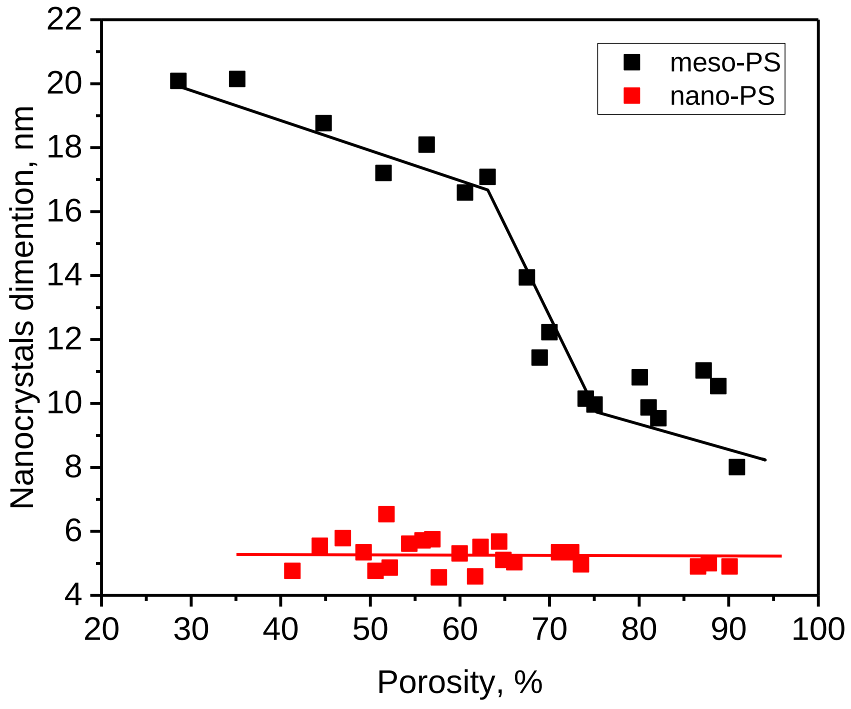

Figure S5. Dimentions of silicon nanocrystalls as function of porosity for the meso- and nano-PSi powders

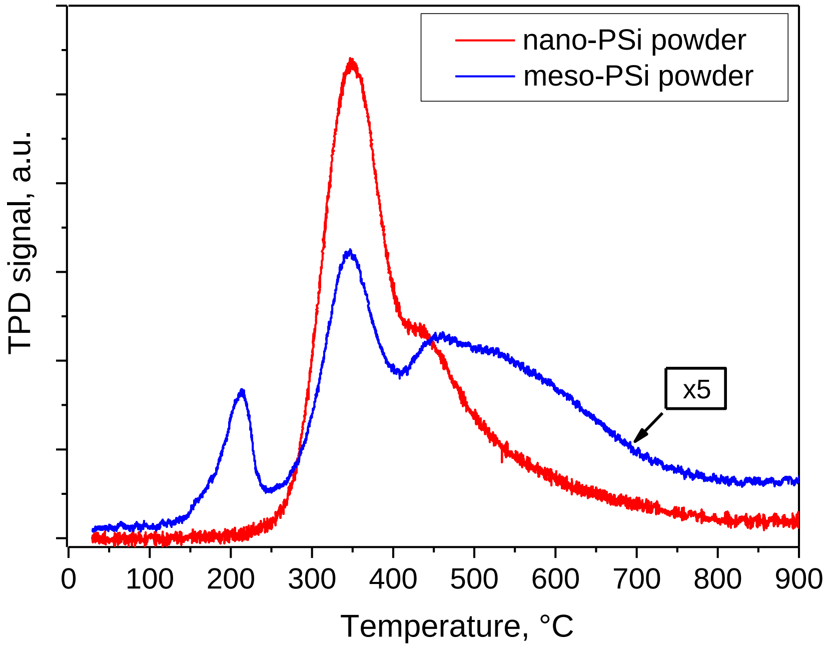

Figure S6. Effusion curves for H_2 desorption from the nano- and meso-PSi powders.

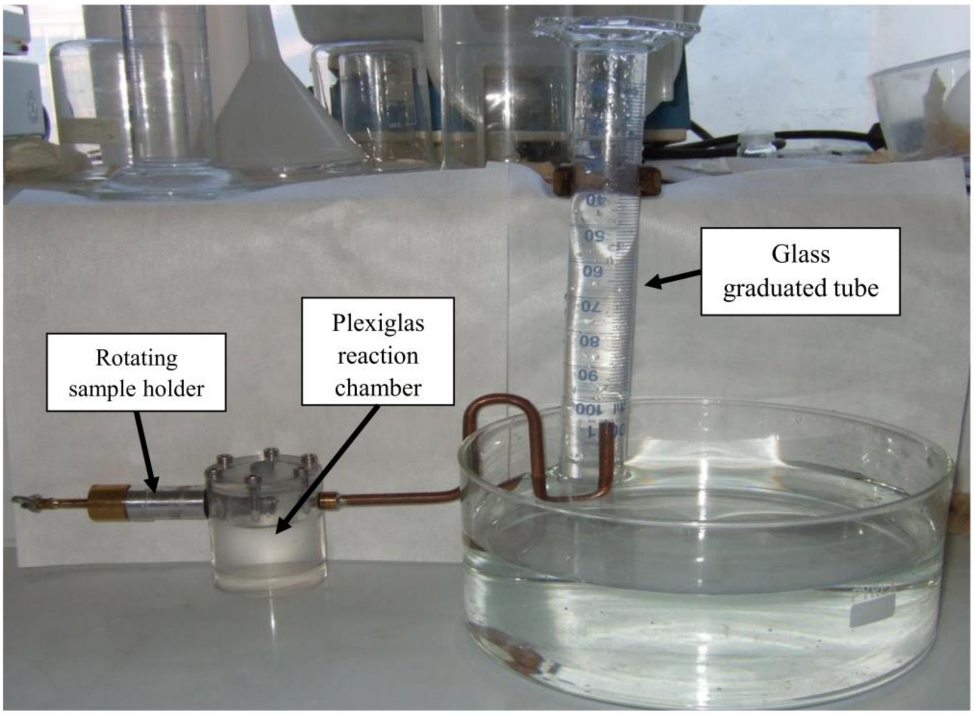

Figure S7. Photo of the working system used for measurements of hydrogen generation kinetics.

Fitting functions

Fitting of experimental points shown in **Figure 4** in the main body of the manuscript have been done with the following futhenction for both -40 ºC and +23ºC:

$$y = y_0 + A_1 e^{-\frac{x}{t_1}} + A_2 e^{-\frac{x}{t_2}} + A_3 e^{-\frac{x}{t_3}} \qquad \text{(S1)}$$

Fitting of experimental points shown in **Figure 5** in the main body of manuscript have been done with the following functions:

In case of NaOH and NH_4OH: $y = y_0 + A_1 e^{-\frac{x}{t_1}} + A_2 e^{-\frac{x}{t_2}} + A_3 e^{-\frac{x}{t_3}}$

In case of KOH: $y = e^{a+\frac{b}{x+c}}$

Fitting of experimental points shown in **Figure 6** in the main body of manuscript have been done with the following functions:

0.13 moles $y = e^{a+\frac{b}{x+c}}$

0.25 moles $y = e^{a+\frac{b}{x+c}}$

0.51 moles $y = a(1 - b^x)$

1.02 moles $y = y_0 + A_1\left(1 - e^{-\frac{x}{t_1}}\right) + A_2\left(1 - e^{-\frac{x}{t_2}}\right) + A_3\left(1 - e^{-\frac{x}{t_3}}\right)$

Fitting of experimental points shown in **Figure 7** in the main body of manuscript have been done with the following functions**:**

As-prepared meso-PSi nanopowder: $y = y_0 + A_1 e^{-\frac{(x-x_0)}{t_1}} + A_2 e^{-\frac{(x-x_0)}{t_2}}$

Grinded meso-PSi nanopowder : $y = y_0 + A_1 + A_2\left(1 - e^{-\frac{x}{t_2}}\right) + A_3\left(1 - e^{-\frac{x}{t_3}}\right)$

Fitting of experimental points shown in **Figure 8** in the main body of manuscript have been done for both p++ c-Si C and p c-Si substrates with the following function:

$$y = y_0 + A_1\left(1 - e^{-\frac{x}{t_1}}\right) + A_2\left(1 - e^{-\frac{x}{t_2}}\right) + A_3\left(1 - e^{-\frac{x}{t_3}}\right)$$

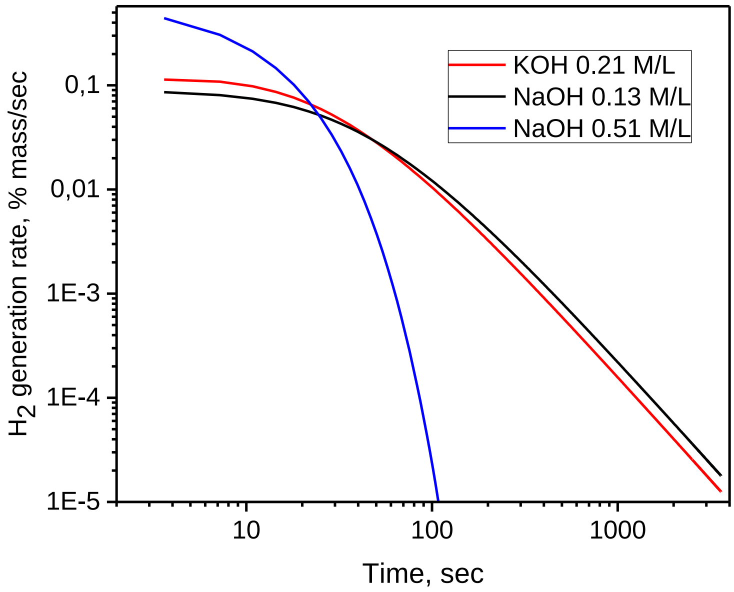

Figure S8. Hydrogen generation rates deduced from experimental the curves shown in Figures 5 and 6.

Effect of PSi nanopowder amount on overall hydrogen generation is shown in Figure S9. As one can see, in order to ensure stable generation of hydrogen one has to work in excess of the oxidizing solution. Volume of the oxidizing solution should be sufficient to completely surround PSi nanoparticles.

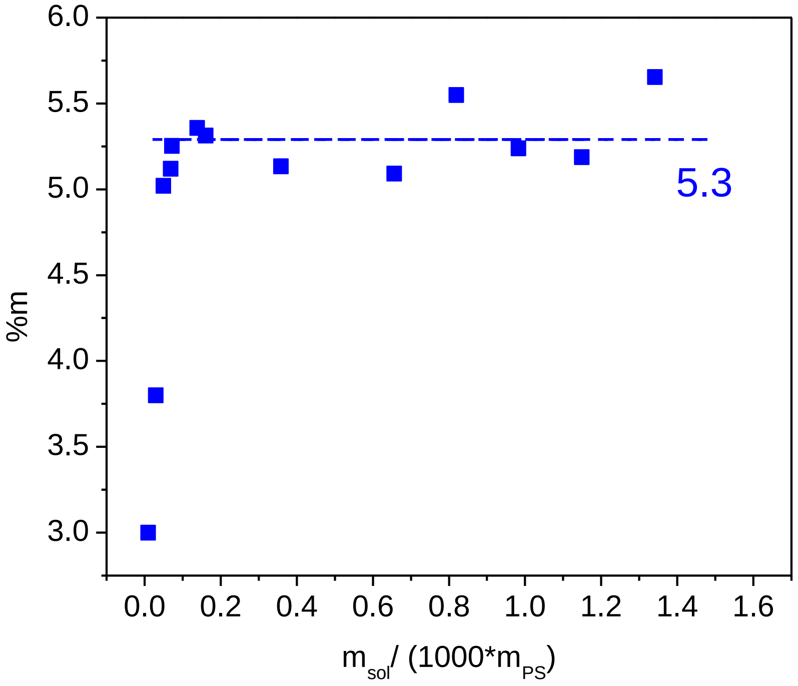

Figure S9. Hydrogen amount in terms of %mass (%m) versus the ratio between mass of the oxidizing solution (m_{sol}) and mass of meso-PSi nanopowder (m_{PS}).

Table S2. Data summarizing the chemical reactions used to produce H_2.

**Reaction**	**H_2, %mass**	**Comment**
$Si + 2H_2O = SiO_2 + 2H_2$.	6.25	Relatively high quantity of H_2. Controllable reaction rate. Nanostructured morphology of PSi allows to avoid surface passivation and to apply low concentrations of alkali catalyst.
$LiAlH_4 + 2H_2O = LiAlO_2 + 4H_2$.	10.81	Very fast uncontrollable exothermic reaction, risk of explosion.
$MgH_2 + H_2O = MgO +2H_2$.	9.09	In fact, $Mg(OH)_2$ is formed. Slow reaction due to surface passivation, unavailability of the material with high surface area.
$CaH_2 + H_2O = CaO +2H_2$.	6.67	Fast uncontrollable exothermic reaction, risk of explosion.
$2Al + 3H_2O = Al_2O_3 + 3H_2$.	5.56	In fact, $Al(OH)_3$ is formed. Passivation of Al surface, high concentration of alkali or other catalyst is needed to complete the reaction.
$Mg + H_2O = MgO + H_2$.	4.76	In fact, $Mg(OH)_2$ is formed.
